# A clearer vision: unveiling the importance of cycloplegic refraction and the pseudomyopia prevalence in Chinese preschoolers

**DOI:** 10.1186/s12886-024-03551-1

**Published:** 2024-08-12

**Authors:** Peipei Liu, Bidan Zhu, Jing Fu, Yunyun Sun, Xiangxiang Liu, Lei Li, Shana Wang, Xi Qin

**Affiliations:** 1 Beijing Tongren Eye Center, Beijing Tongren Hospital, Capital Medical University, Beijing Ophthalmology & Visual Sciences Key Laboratory, Beijing, China; 2 Tongzhou Maternal and Child Health Hospital of Beijing, Beijing, China

**Keywords:** Cycloplegic, Refraction, Pseudomyopia, Preschool children, COVID-19

## Abstract

**Background:**

This study aimed to investigate the difference between cycloplegic and noncycloplegic refraction and evaluate the pseudomyopia prevalence in Chinese preschool children during the outbreak of COVID-19.

**Methods:**

A cross-sectional study was conducted in the Tongzhou District of Beijing, China. Refractive error was measured under both noncycloplegic and cycloplegic conditions with autorefraction. The difference between noncycloplegic and cycloplegic spherical equivalent refraction (SER) and pseudomyopia prevalence were analyzed. Pseudomyopia was defined as SER ≤-0.50D in precycloplegic assessments and >-0.50D in post-cycloplegic assessments.

**Results:**

Out of the 1487 participants who were enrolled in the study, 1471 individuals (98.92%) between the ages of 3–6 years completed all required procedures. A statistically significant difference in refraction was observed between noncycloplegic and cycloplegic measurements, the median of difference in spherical equivalent refraction (SER) of 0.88D (dioptre)(0.50,1.38). There was a high intraclass correlation (ICC) between these two methods for cylinders (ICC = 0.864; 95% CI, 0.850–0.877). The median DSE for myopia, emmetropia and hyperopia were 0.25D (0.00, 0.38),0.25D (0.06, 0.50) and 1.00D (0.62, 1.38), an hypermetropes showed considerably greater differences than myopes and emmetropes (Kruskal-Wallis test, H = 231.023, *P* = 0.000). Additionally, girls displayed a greater DSE than boys. Furthermore, when comparing against-the-rule (ATR) and oblique astigmatism, it was found that with-the-rule (WTR) astigmatism had the largest DSE. The study found varying prevalence rates of myopia, emmetropia, and hyperopia with and without cycloplegia, which were 1.90% vs. 10.06%, 11.49% vs. 50.31%, and 86.61% vs. 39.63%, respectively. Additionally, the overall prevalence of pseudomyopia was determined to be 8.29%. Participants with pseudomyopia had a significantly higher mean difference in SER (DSE) compared to non-pseudomyopic participants.

**Conclusions:**

Cycloplegic refraction is more sensitive than a noncycloplegic one for measuring refractive error in preschool children. Pseudomyopia is prevalent in preschool children during the COVID-19 outbreak period. Our study indicates the possibility that cycloplegic refraction should be performed in preschool children routinely.

## Introduction

Pseudomyopia is a phenomenon that occurs due to accommodation, particularly in children, resulting in an apparent myopic refractive error [1]. When cycloplegia is administered, it can reduce or even eliminate the condition. There is a limited amount of research available on pseudomyopia, with reported incidence rates ranging from approximately 8% in a study conducted in Beijing [2] to 24.1% in a study conducted in Anyang [3]. Pseudomyopia is diagnosed and measured by the difference in spherical equivalent refraction (DSE) under fully cycloplegic and non-cycloplegic conditions [1]. Mei and Rong [4]reported that pseudomyopia is a transient stage before permanent myopia developed, and it usually develops after sustained near-work. Excessive near-work was thought to mediate the association between myopia progression and increased accommodation [5, 6].

Beijing has been affected by multiple waves of COVID-19 outbreaks. To effectively control this outbreak, local authorities have urged people not to move freely to contain the spread of the virus by implementing travel bans, lockdowns, and quarantines [7], which could lead to decreased time spent outdoors, and more sustained near-work and digital screen time. These forced behavioral changes may, therefore, have an effect on the refractive state of the children, including worsening myopia or causing more pseudomyopia cases. As a result of these changes in behavior, an unwelcome but relevant opportunity may be offered to examine the changes in refractive states [8].

Among pediatric populations, cycloplegic refraction is the gold standard for estimating refractive error [9].Astigmatism showed high diagnostic agreement when noncycloplegic measures were used, but other refractive errors showed low diagnostic agreement. In Beijing, there are few studies examining the systematic differences in refractive error measurements between preschool-aged individuals with and without cycloplegia. Most studies have addressed the refractive error of school-aged children and adults [3, 10, 11], with few studies conducted among preschool-aged children [12, 13], especially after the COVID-19 pandemic, and the prevalence of pseudomyopia in this group has hardly been reported. Considering young children may be more susceptible to environmental changes than those of older children [14], it is imperative to update recent data on refractive error prevalence. The long-term aim of this study is to assess the potential impact of COVID on refractive status in preschoolers in Beijing and explore possible relationships between pseudomyopia and myopia onset/progression. Two aims are presented in this paper. The first is to compare refractive status before and after cycloplegic in Tongzhou District of Beijing, China. A second aim is to analyze the age-specific prevalence of pseudomyopia in preschool children.

## Methods

### Study population

An ongoing prospective study has been performed annually on participants from 9 kindergartens in Tongzhou district, Beijing, China, since 2021, and students were screened from December to January. The project was approved by the Beijing Tongren Hospital Ethical Committee, following the Declaration of Helsinki. Written consent was obtained from the parent of each participant, as well as verbal consent from each participant. There was no compensation or incentive offered for participation. This manuscript analyzed the baseline cross-sectional data of this study(Data collected between December 2021 and January 2022), aiming to evaluate prevalence of refractive error in preschool children during the COVID-19 outbreak. In this study, participants’ ages are based on their screening date. The study methodology followed the Strengthening the Reporting of Observational Studies in Epidemiology (STROBE) reporting guideline. Exclusion criteria were subjects with ocular abnormalities; a history of ocular surgery or trauma; and systemic diseases that may affect vision. A total of 1515 participants from 9 kindergartens in Tongzhou District were included. 28 of these participants were excluded according to the criteria. Therefore, 1487 participants were enrolled in this study.

### Procedures

All participants received comprehensive ophthalmic examinations. Visual acuity at distance and near, dominant eye(by “the hole-in-a-card” test [15]), intraocular pressure, slit lamp biomicroscopy, non-cycloplegic and cycloplegic refraction, Hirschberg test, alternate cover test, ocular motility, and a lenstar LS900 (HAAG-STREIT AG, Switzerland) was used for ocular biometry. Refractive errors before and after cycloplegia were measured with a calibrated autorefractor (KR-800, Topcon). Measurements were repeated three times and averaged. For both cylinders and spheres, the readings must be at most 0.50D apart. Whenever this was not possible, the measurements were repeated [13]. As young participants with dark iris have difficulty achieving adequate cycloplegia, 1% cyclopentolate was used to accurately measure refractive error [14]. To alleviate discomfort, participants were first administered one drop of Alcaine (Proparacaine Hydrochloride 0.5%, Alcon). Afterwards, apply two drops of 1% cyclopentolate (Alcon) and one drop of Mydrin P (Tropicamide 0.5%, Phenylephrine HCl 0.5%; Santen Pharmaceutical, Japan) after a 5-minute interval. Eyes were examined after 30 min for pupil size and pupil response to light. If the pupil did not dilate and there was no response to light, the eye was classified as cyclopleged. If necessary, an additional drop may be administered.

### Definitions

SER was defined as the sum of the spherical power and half the cylindrical power. Pseudomyopia was defined as SER ≤ − 0.50 D before cycloplegia and > − 0.50D after cycloplegia [3]. DSE was calculated as cycloplegic SER minus non-cycloplegic SER. Emmetropia was defined as SER between − 0.5 D and + 0.5 D. Myopia was defined as SER ≤ − 0.50 D, and hyperopia as SER ≥ + 0.50 D. There are three categories of hyperopia: low (+ 0.5 D ≤ SER < + 2.0 D), moderate (+ 2.0 D ≤ SER < + 5.0 D) and high (SER ≥ + 5.0 D) [16].

### Statistical analysis

Statistical analyses were performed using SPSS software (version 22.0 SPSS; Chicago, IL, USA). Because of the high correlation coefficient (Pearson correlation coefficient = 0.90) between cycloplegic SER in the two eyes, only the right eyes were analyzed. Continuous variables were presented as mean ± standard deviation. The data on refractive error did not follow a Gaussian distribution, and were presented as a median and interquartile range. TheWilcoxon signed rank test andKruskal-Wallis test were used to explore the DSE and SER between different groups.χ2 tests were used to compare the prevalence of pseudomyopia and refractive errors among different groups. The agreement between cycloplegic and noncycloplegic refraction was analyzed using intraclass correlation analysis (ICC) and Bland-Altman analysis. The ICC value less than 0.5 indicates poor reliability, the value between 0.5 and 0.75 indicates moderate reliability, the value between 0.75 and 0.9 indicates good reliability, and the value over 0.90 indicates excellent reliability [17]. The *P* values were all two-sided and statistically significant when 0.05 or less.

## Results

Among 1487 participants examined in this study, 1471 (98.92%) who completed a baseline examination were included in the analysis. The children’s mean age was 4.42 ± 0.83 years (range, 3–6), and 776 (52.75%) were boys. Noncycloplegic SER ranged from − 10.13 D to 5.75D and the cycloplegic SER ranged from − 8.38D to 6.88D.

**Fig. 1:**
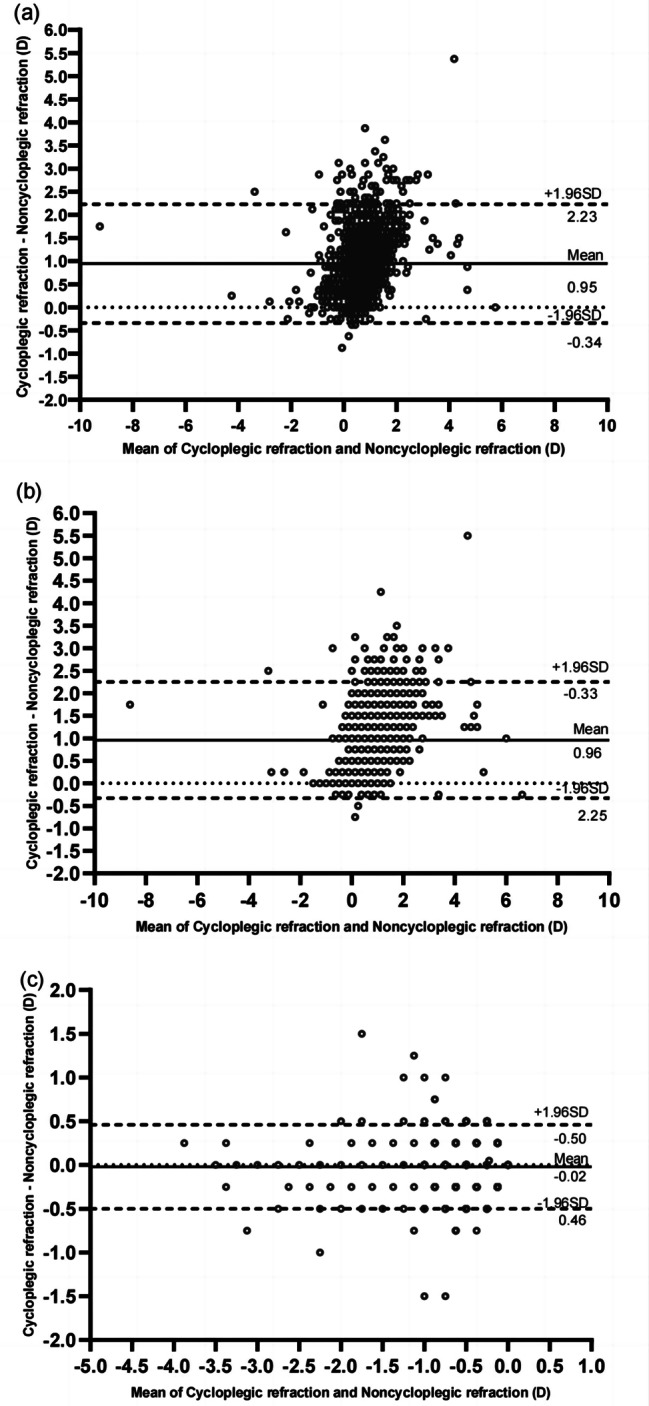
Bland-Altman plots illustrating the comparison of means and differences between cycloplegic and noncycloplegic refraction of the spherical equivalent **(a)**, sphere **(b)**, and cylindrical values **(c)**. The central solid line denotes the average difference between the two measurements, while the limits of agreement (LoA) are delineated by the dashed lines at the upper and lower boundaries of the graph

**Fig. 2:**
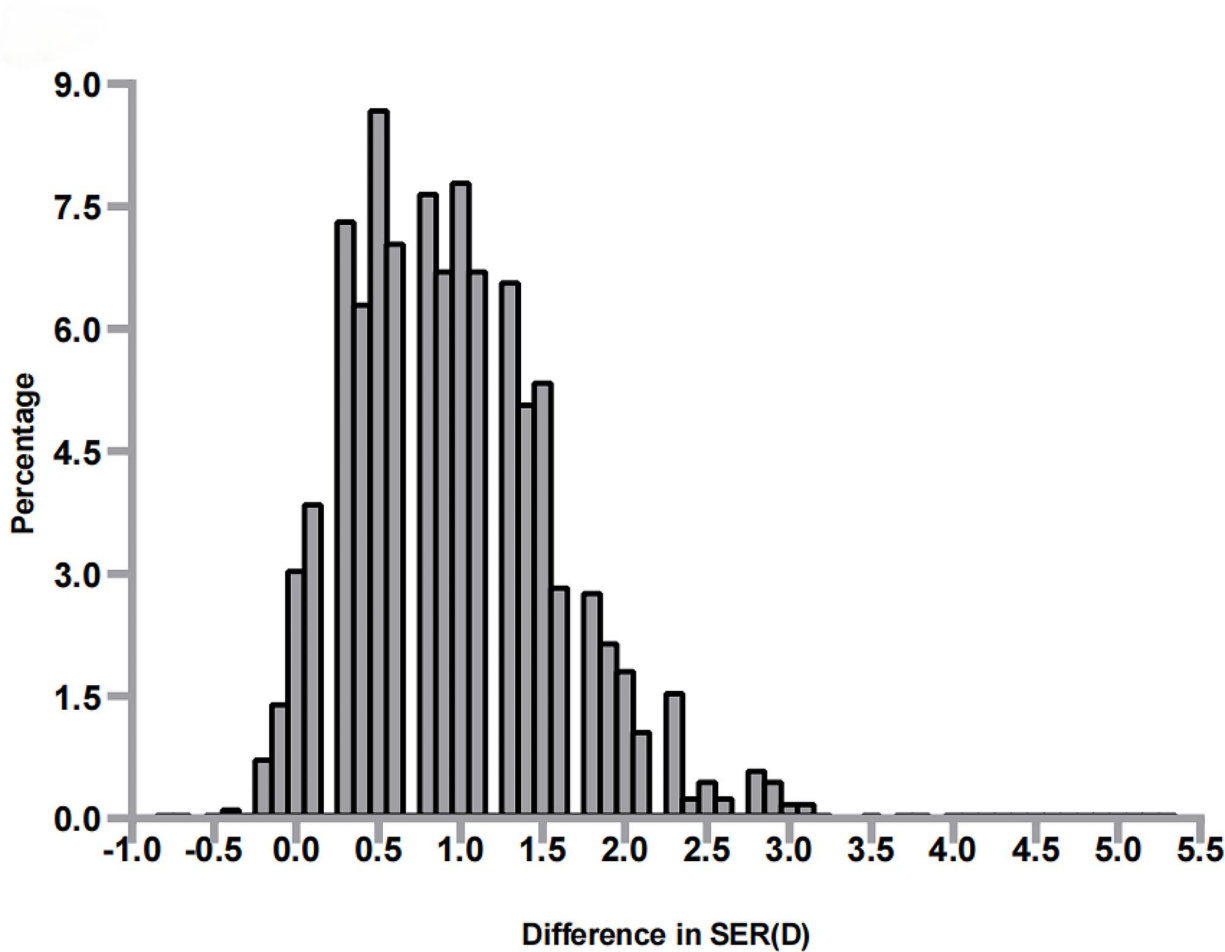
The distribution of difference in SER for all participants. SER, spherical equivalent refraction

The median spherical value measured 0.50D (0.25, 0.75), and cylindrical value were-0.50 D(-0.50, -0.25) before cycloplegia. After cycloplegia, the values were 1.50D (1.00, 1.75) and − 0.50D (-0.75, 0.25), with statistically significant differences in the spherical values (Wilcoxon signed rank test, T = 967,046, *P* = 0.000). The intraclass correlation coefficients (ICC) for cylinder between cycloplegic and noncycloplegic refraction were found to be high (ICC = 0.864; 95% CI, 0.850–0.877).This indicates good reliability. while the ICC values for sphere and SER were 0.406 (95% CI, -0.095–0.704) and 0.403 (95% CI, -0.095–0.700), indicating poor reliability. The Bland-Altman plot (Fig. 1) illustrates the mean differences between cycloplegic and noncycloplegic refraction for sphere (0.96 ± 0.66D, 95% LoA: −0.33 to 2.25D), cylinder (− 0.02 ± 0.24D, 95% LoA: −0.50 to 0.46D), and SER (0.95 ± 0.66D, 95% LoA: −0.34 to 2.23D). The median non-cycloplegic SER was 0.38D (0.00, 0.63), while the median for cycloplegic SER was 1.25D (0.75-1.63D), and statistically significant difference was found between them(Wilcoxon signed rank test, T = 1013452.00, *P* = 0.000). A comparison of cycloplegic and noncycloplegic refractions is shown in Table 1. After cycloplegia, the total prevalence of myopia decreased from 10.06 to 1.9%, and emmetropia prevalence decreased from 50.31 to 11.49%, as for hyperopia, it increased from 39.63 to 86.68% (*P*<0.01 for all). It was found that noncycloplegic refraction tends to overestimate myopia by 81.67% and underestimate hyperopia by 47.05% when compared to cycloplegic refraction. 82.43% (122/148) of participants initially diagnosed as myopic prior to cycloplegia exhibited emmetropic or hyperopic refractive errors post-cycloplegia, while 77.16% (571/740) of participants initially diagnosed as emmetropic prior to cycloplegia were found to have hyperopic refractive errors post-cycloplegia. Noncycloplegic refraction exhibited less positive (more negative) results than cycloplegic refraction with the median difference of 0.88D (0.50, 1.38)(Fig. 2).

**Table 1:** Prevalence rates of refractive error before and after cycloplegia

	Noncycloplegic %(95%CI); *n*	Cycloplegic %(95%CI); *n*	χ^2^	*P*
Myopia				
-1.50D< SER≤-0.50 D	8.70(7.37–10.25); 128	1.36(0.88–2.09); 20	82.985	0.000
-2.50 D< SER≤-1.50 D	1.02(0.62–1.68); 15	0.34(0.15–0.79); 5	5.034	0.025
SER≤-2.50 D	0.34(0.15–0.79); 5	0.20(0.07–0.59); 3	0.125	0.723
All	10.06(8.63–11.70); 148	1.90(1.32–2.73); 28	87.024	0.000
Emmetropia				
-0.50 D< SER<0.50 D	50.31(47.76–52.86); 740	11.49(9.96–13.22); 169	519.055	0.000
Hyperopia				
0.50 D ≤ SER<1.50 D	37.80(35.36–40.31); 556	51.39(48.84–53.94); 756	55.028	0.000
1.50 D ≤ SER<2.00 D	0.88(0.51–1.50);13	21.28(19.26–23.44); 313	310.477	0.000
2.00 D ≤ SER<3.00 D	0.41(0.19–0.89); 6	10.88(9.39–12.57); 160	151.411	0.000
SER ≥ 3.00 D	0.54(0.27–1.06); 8	3.06(2.29–4.07); 45	26.304	0.000
All	39.63(37.16–42.15); 583	86.61(84.77–88.26); 1274	697.200	0.000

CI confidence interval, D dioptre, *n* number of participants, SER spherical equivalent refraction

After being divided into different groups, the DSE for myopia, emmetropia and hyperopia was 0.25D(0.00, 0.38), 0.25D(0.06, 0.50) and 1.00D(0.62, 1.38), respectively (Kruskal-Wallis test, H = 231.023, *P* = 0.000). Hypermetropes showed considerably greater differences than myopes and emmetropes. Among different astigmatism types, the DSE for oblique, against-the-rule (ATR) and with-the-rule (WTR) was 0.75D (0.38,1.25), 0.75D (0.38–1.25), and 1.00D (0.5,1.38) (Kruskal-Wallis test, H = 14.236, *P* = 0.001), that WTR astigmatism presents the largest DSE.

For all participants, the median DSE for girls was 0.88D(0.50, 1.38), which was higher than boys 0.88D(0.50, 1.25) (Kruskal-Wallis test, H = 5.059, *P* = 0.024). There is no significant variation in DSE across different age groups or among individuals with varying cylindrical values.

The prevalence of refractive error in different groups, determined by cycloplegic autorefraction, is shown in Table 2. Among the 3- to 6-year-old participants, low hyperopia was predominant (72.67%), while myopia was uncommon (1.9%). The prevalence of myopia in 3-year-old children was 3.2%, which was slightly higher than in other age groups, although this difference was not statistically significant. The prevalence of emmetropia was similar in participants aged 3 to 5 years. Although the 6-year-old participants had the lowest value, it was not statistically different. However, there was a significant difference in hyperopia prevalence across age groups (*P*<0.05). The proportions of low hyperopia for participants 3–6 years were 69.86%, 70.12%, 73.74%, and 84.62%, respectively, while the proportions of moderate and high hyperopia were15.07%, 16.99%, 12.26%, and 6.73%, respectively. The prevalence of refractive errors did not differ statistically significantly between between the sexes.

**Table 2:** Prevalence of refractive errors among 3− to 6−year−old participants determined by cycloplegic autorefraction

		Myopia			Emmetropia			Low hyperopia			Moderate and high hyperopia		
		(SER ≤ ^−0.50D)^			(-0.50D<^SER^<^0.50D)^			(0.50D ≤ ^SER^<^2.00D)^			(SER ≥ ^2.00D)^		
Variable	Number	Number % 95% CI			Number % 95% CI			Number % 95% CI			Number % 95% CI		
Age(years)													
3	219	7	3.2	1.56–6.45	26	11.87	8.23–16.82	153	69.86	63.48–75.55	33	15.07	10.94–20.41
4	512	8	1.56	0.79–3.05	58	11.33	8.87–14.37	359	70.12	66.02–73.92	87	16.99	13.99–20.49
5	636	11	1.73	0.97–3.07	78	12.26	9.94–15.04	469	73.74	70.18–77.01	78	12.26	9.94–15.04
6	104	2	1.92	0.53–6.74	7	6.73	3.3-13.24	88	84.62	76.47–90.31	7	6.73	3.30-13.24
^χ2^		2.382			2.736			10.389			10.205		
*P* ^value^		0.497			0.434			0.016			0.017		
Sex													
Male	776	13	1.68	0.99–2.85	96	12.37	10.24–14.87	569	73.32	70.10-76.31	98	12.63	10.48–15.15
Female	695	15	2.16	1.31–3.53	73	10.5	8.43-13.00	500	71.94	68.49–75.15	107	15.4	12.91–18.27
χ2		0.458			1.257			0.353			2.34		
*P* ^value^		0.499			0.262			0.553			0.126		
All	1471	28	1.9	1.32–2.73	169	11.49	9.96–13.22	1069	72.67	70.34–74.89	205	13.94	12.26–15.80

CI confidence interval, D dioptre, SER spherical equivalent refraction

**Fig. 3:**
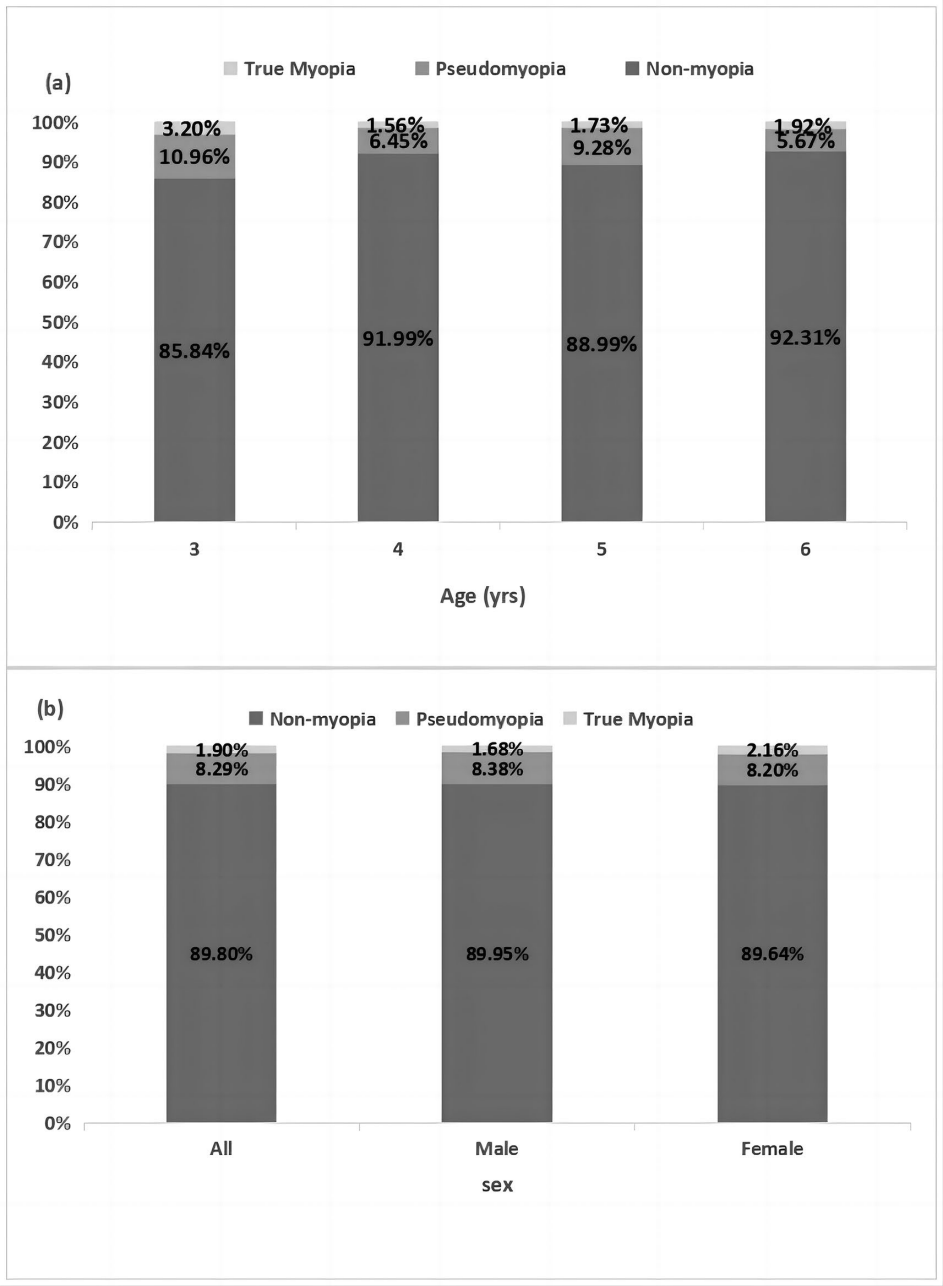
**(a)** Distribution of refractive status by age. **(b)** Distribution of refractive status by sex. Non-myopia: both noncycloplegic refraction and cycloplegic refraction>-0.50 D; Pseudomyopia: noncycloplegic refraction ≤-0.50 D and cycloplegic refraction >-0.50 D; True myopia: cycloplegic refraction ≤-0.50 D

Figure 3 shows the percentage of the different refractive categories in each age and sex group. There were 122 with pseudomyopia. These accounted for 8.29% of all participants, and the prevalence rate of males and females was similar. The proportion of pseudomyopia decreases with age. Pseudomyopia was less prevalent in 6-year-old participants than in 3-year-olds (5.67% vs. 10.96%), the prevalence of pseudomyopia among different age groups was not statistically significant, however. Statistically significant DSE was found between pseudomyopia and non-pseudomyopia (True myopia + Non-myopia), participants with pseudomyopia having more hyperopic DSE than non-pseudomyopia(Kruskal-Wallis test, H = 70.709, *P* = 0.000). In pseudomyopia participants, DSE of different age groups is similar.

**Table 3:** Studies comparing noncycloplegic and cycloplegic refraction in children

References	Location	Year	Mean age	Cycloplegia	*n*	DSE(D)	Age group	DSE(D)
			(years)					
present	Beijing	2021	4.42 ± 0.83(range, 3–6)	two drops of 1% cyclopentolate and one drop of Mydrin P	1471	0.88(0.50,1.38)	3	0.88(0.50,1.38)
							4	1.00 (0.50,1.38)
							5	0.88(0.50,1.25)
							6	0.88(0.50,1.22)
Li et al. [10]	Tibetan Plateau	2019	6.83 ± 0.46(range,5.9–10.3)	two drops of 1% cyclopentolate and one drop of Mydrin P	1830	0.90 ± 0.76	6	0.89
							7	0.98
							8	0.73
Hu et al. [11]	Shandong	2015	10 ± 3.3(range, 4–18)	Three drops of 1% cyclopentolate	5999	0.78 ± 0.79		
Kang et al. [3]	Anyang	2018 or before	6.5 ± 0.5 and 13.0 ± 0.5	Two drops of 1% cyclopentolate and one drop of tropicamide	2612(6-year-olds)	-	6	1.13(0.63,1.63)
					1984(13-year-olds)		13	0.38(0.13,0.88)
Lin et al. [2]	Beijing	2010	10.8 ± 3.2(range, 6–17)	Three drops of 1% cyclopentolate	219	0.51 ± 0.72		
Sankaridurg et al. [12]	Shanghai	2017	9.1 ± 2.9(range, 4–15)	Two or three drops of 1% cyclopentolate	6017	0.63 ± 0.65	4	0.95
							5	0.89
							6*	0.89
Zhu et al. [18]	Inner Mongolia	2013	11.9 ± 3.5(range, 6–21)	Two or three drops of 1% cyclopentolate	1565	0.57		
Fotouhi et al. [19]	Tehran	2002	31.6 ± 18.11 (range, 5–95)	Two drops of 1% cyclopentolate	3501	0.71**		
Fotedar et al. [20]	Sydney	2005 or before	12 and 6	One drop of 1% cyclopentolate	210(6-year-olds)	-	6	1.18
					2233(12-year-olds)		12	0.84
Guo et al. [21]	Chicago	2016–2019	10.8 ± 4.0(range, 3–22)	One drop of 1% tropicamide and 2.5% phenylephrine and 2 drops of 1% cyclopentolate	11,119	0.65 ± 1.04		

*The results were not listed participants aged 7 to 15 years. **The DSE was the participants group aged 5–10

*n* number of participants, D dioptre, DSE the difference in spherical equivalent refraction

## Discussion

The current research revealed that pseudomyopia was present in 8.29% of preschool children in the Tongzhou District of Beijing, China. The predominant refractive error among preschool children was mild hyperopia (72.67%), with a prevalence of myopia at 1.9%. Additionally, this study investigated the differences in refraction between noncycloplegic and cycloplegic conditions. The results indicated that noncycloplegic refraction was, on average, more myopic than cycloplegic refraction, with hyperopic participants experiencing the greatest deviation and myopic children showing the smallest deviation. Furthermore, the findings of this study indicate that assessing the refractive status of preschool children without the use of cycloplegia can lead to substantial inaccuracies, including underestimation of hyperopia prevalence and overestimation of myopia and emmetropia.

The median of cycloplegic SER in the present study was 1.25D (0.75-1.63D). These findings are in line with other published studies conducted on preschool-age participants. In the Guangzhou study, the mean cycloplegic SER was 1.42 ± 0.79D [14]. Two additional studies had similar results, with 1.20 ± 1.05D in the Shanghai study [22] and 1.38 ± 0.73 D in the Shenzhen study [23]. In a Singapore study, cycloplegic SER for participants aged 3–5 were1.14 ± 0.73 D, 1.38 ± 0.88 D, and 0.97 ± 0.90 D [24], respectively. Studies comparing noncycloplegic and cycloplegic refraction in children are listed in Table 3. We also compared the differences under cycloplegic and non-cycloplegic refraction conditions. After being divided into different groups according to their refractive states (myopia, emmetropia, and hyperopia), the DSE of hyperopic participants was greater than that of emmetropic and myopic participants, and comparable to previous results [2, 10, 12, 20]. In our study, the DSE of boys was lower than that of girls for all participants and consistent with prior findings [10, 11, 20]. Some studies, however, found no statistically significant association between the DSE and sex [2, 11]. Furthermore, an examination of the correlation between astigmatism and DSE revealed that WTR astigmatism exhibited an association with larger DSE, contrasting with the findings of the Lhasa Childhood Eye Study (LCES) [10]. Studies have also found that DSE decreases with age [3, 9, 11]. It is worth noting that our current study did not observe this pattern. Such inconsistencies could potentially be attributed to variations in environmental factors, age demographics, ethnic backgrounds, and lifestyle practices. Additional research endeavors are warranted to delve deeper into this relationship.

In the current study, low hyperopia was the most common refractive error(>70%), this finding was similar to that of previous studies [21, 23, 25] There was an overall prevalence of 1.9% of myopia in this study. In comparison with studies conducted in Shenzhen(1.3%) [23] and Guangzhou(1.0%) [14], a higher prevalence was found in the current study. As compared with that reported in studies conducted in Shanghai (3.7%) [22] and Singapore (> 6%) [24], the current study found a relatively lower prevalence. In summary, myopia was still very rare in this preschool population. It differed from several previous studies [13, 26, 27]. A majority of these published studies focused on school-aged children and adolescents. Due to cycloplegic methods, investigation time points, and home confinement regulations, the results of our study cannot be compared directly with those of previous studies. A Taiwanese study reported that the prevalence of myopia did not vary significantly under COVID-19 social restrictions in preschool-aged children [28], which is similar to the present study. There may be a couple of explanations for this. First, preschool-aged children have less dependence on computers and smartphones, and their screen time is also less than that of school-aged children [29]. Second, a few months of social restrictions might not be long enough to trigger myopia shift in preschoolers [28], as they often have a greater emmetropisation reserve [3]. The prevalence of refractive errors was similar between the sexes in our study, similar to the Singapore study [24] and Shenzhen study [23].The result was inconsistent with other studies; Shanghai [22] and Guangzhou [14] studies reported that boys had an ascending trend of myopia prevalence while girls had no this trend. Different studies have found a different gender effect on myopia prevalence in preschool children, possibly due to environmental factors such as near-work activities and education. Although the difference in the prevalence of myopia among different age groups was not statistically significant, 3-year-olds showed the highest prevalence in our study. Younger participants may be more sensitive to environmental change than older children [13]: more research is required.

The value of noncycloplegic assessments of the eye’s refractive state is limited. The noncycloplegic evaluation of refractive error tends to overestimate myopia and introduces a higher margin of error in the assessment of hyperopic and emmetropic refractive errors. Noncycloplegic measurements lead to incorrect refractive error classification. Thus, cycloplegia plays a crucial role in research on preschoolers’ refraction [30]. A survey in Anyang [3] found that many opticians still lack theoretical knowledge about the right age for cycloplegia, and there is very little recognition of the need for cycloplegia. Therefore, it is necessary to raise knowledge and awareness about the appropriate use of cycloplegia in children.

Pseudomyopia is a condition in which there is an increase in refractive power due to accommodation, it disappears with cycloplegia, and the affected eye is actually emmetropia or hyperopia. Its characteristics at different ages are not well understood. The hospital-based study tended to enroll more myopic children; thus, there is little information on the nature distribution of pseudomyopia [3]. In our study, the prevalence of pseudomyopia was 8.29%, which is consistent with the Beijing Myopia Progression Study [2]. Kang et al [3]investigated the prevalence of pseudomyopia in 6- and 13-year-old children and found that it was 18.9% and 24.1%, which was higher than that in our study. There might be an explanation for such inconsistency: the age of the subjects varied considerably among different studies, and the pseudomyopia rate may varied among different years and regions. Moreover, this study was conducted during the COVID-19 outbreak in China, while other studies reveal pre-COVID-19 data. Compared with non-pseudomyopic children, the pseudomyopic children had higher DSE. Kang et al. [3]. reported that the median DSE of 6-year-old pseudomyopia children was 1.13 D (0.63, 1.63). This result is significantly lower than that obtained in the present study, which is 2 D (1.63, 2.3). In light of the close relationship between pseudomyopia and accommodation [1], the possible reason is increased accommodation due to more intense near-work or screen time during the outbreak [31]. In addition, the prevalence of pseudomyopia was similar between the sexes, and the DSE was overall similar for males and females. These results might suggest that accommodation was not significantly different between sexes.

This study is subject to several limitations. Firstly, the auto-refractometer utilized is a commonly employed tool in epidemiological research due to its convenience and reliability. However, the accuracy of autorefraction may be influenced by children’s compliance during the examination and their visual behavior. To mitigate potential sources of interference, multiple measurements were taken and abnormal values were reevaluated. Secondly, the baseline data in this study is cross-sectional, precluding the ability to establish a causal relationship between pseudomyopia and the onset or progression of myopia. Therefore, longitudinal analysis should be conducted, with future research focusing on potential differences in myopia prevalence during and post-COVID. Additionally, it should be noted that the uneven distribution of participants across age groups, particularly with fewer cases in the 6-year-olds and 3-year-olds, is a common issue in longitudinal studies that may impact results and warrants further investigation. Furthermore, all study participants exhibited light to dark-brown iris colors, which is important to consider given the potential impact of iris color on the effectiveness of cycloplegic drugs. Caution should be exercised when extrapolating these findings to individuals with different iris colors. In conclusion, the present study provides definitive information about the prevalence of refractive error and pseudomyopia in preschool children in China during the epidemic period. Most of the preschool children in Beijing in this study have low hyperopia, the prevalence of myopia is relatively low, and pseudomyopia is more common. The lack of cycloplegia can lead to significant misclassifications of hyperopia, emmetropia, and myopia. Considering that cycloplegic agents can eliminate pseudomyopia, it is necessary to improve the knowledge and awareness about the proper use of cycloplegic agents. Cycloplegia refraction is essential in studies of refractive error in preschool children.

## Data Availability

The article includes all relevant data. Upon reasonable request, data can be obtained. Please contact the corresponding author for further details.
